# Metastases can occur in cirrhotic livers with patent portal veins

**DOI:** 10.1186/s13000-021-01076-5

**Published:** 2021-02-27

**Authors:** Zaid Mahdi, Mark G. Ettel, Raul S. Gonzalez, John Hart, Lindsay Alpert, Jiayun Fang, Natalia Liu, Suntrea T. Hammer, Nicole Panarelli, Jerome Cheng, Joel K. Greenson, Paul E. Swanson, Maria Westerhoff

**Affiliations:** 1 Department of Pathology, Emory University School of Medicine, Atlanta, GA USA; 2 Department of Pathology, University of Rochester Medical Center, Rochester, NY USA; 3 Department of Pathology, Beth Israel Deaconness Medical Center, Boston, MA USA; 4 Department of Pathology, University of Chicago, Chicago, IL USA; 5 Department of Pathology, University of Michigan, Faculty Suite Rm. 36-1221-65 2800 Plymouth Rd, Building 35, 48109 Ann Arbor, MI USA; 6 Department of Pathology, University of Texas Southwestern Medical Center, Dallas, TX USA; 7 Department of Pathology, Montefiore Medical Center, Bronx, NY USA; 8 Department of Pathology, University of Washington, Seattle, WA USA

**Keywords:** Liver, Metastases, Liver mass, Laennec staging, Cirrhosis

## Abstract

**Objectives:**

Metastases are common in non-cirrhotic livers but are considered unlikely in the setting of cirrhosis. However, the degree of fibrosis in cirrhosis may vary; thus metastases may still access the liver vasculature and present as a mass in cirrhotic livers. This possibility may affect pathologists’ diagnostic algorithms when faced with a liver mass biopsy.

**Methods:**

We hypothesized that metastases can occur in cirrhotic livers if fibrous remodeling is not severe or abnormal veno-arterial shunting exists to override an obstructed portal system. We searched departmental archives for cirrhotic livers with masses, categorizing fibrosis by Laennec staging: 4A = mild cirrhosis, 4B = moderate, 4 C = severe.

**Results:**

Of 1453 cirrhotic livers with masses, 1429 were primary tumors and 24 were metastases (1.7 %). Of livers with metastases, most had 4A or 4B cirrhosis by Laennec staging (*n* = 17; 71 %). Eleven patients were evaluated by ultrasound Doppler; 2 of 5 with Laennec 4 C had reversal of portal vein flow, but all 4A & 4B patients had patent portal veins without reversed flow. Echocardiograms (13 patients) showed no ventricular or atrial septal defects or arteriovenous shunts.

**Conclusions:**

Metastases are uncommon in cirrhotic livers, accounting for 1.7 % of masses. Most involved livers had mild or moderate cirrhosis (Laennec 4A/4B) and patent portal veins; however, as some Laennec 4 C cases also contained metastases, obstructed portal access may not be enough to deter metastatic access.

## Introduction

The liver is among the most common sites of metastatic disease. However, while metastases are common in non-cirrhotic livers, multiple autopsy studies have confirmed that they are rare in the setting of cirrhosis.[1–3] As such, pathologists generally regard metastatic malignancy as an unlikely diagnosis when evaluating biopsies from mass lesions in cirrhotic livers.

To explain the frequency of liver metastases in the general population and the contrasting rarity of metastases in the cirrhotic liver, a review of the main hypotheses for development of metastatic disease is appropriate. The “seed and soil” hypothesis, first offered by Stephen Paget in 1889, states that the “seed” of metastatic tumor cells may develop into a full-fledged metastasis only once it reaches the favorable “soil” of a hospitable environment such as the otherwise healthy liver.[4, 5] In contrast, the advanced fibrosis and microenvironment of the cirrhotic liver may be an “unfavorable soil” for metastatic carcinomas. Alternatively, James Ewing hypothesized that hemodynamic factors are responsible for the appearance and distribution of metastases.[4] In non-cirrhotic livers, carcinomas may access the liver through the dominant portal venous blood supply, resulting in a high incidence of liver metastases from gastrointestinal primary malignancies. However, in cirrhosis, there is scarring and increased intrahepatic resistance to blood flow. It is plausible that this scarring may deter the entry of metastases via reversal of portal venous blood flow and development of porto-systemic shunts that bypass the liver.[6, 7] Previous studies show that the hepatic artery is another route for metastatic cells to access the liver.[8, 9].

Contrary to previous dogma, cirrhosis is not an “all or nothing,” irreversible process. There can be a spectrum of fibrosis, and the extent of fibrosis in a given patient likely will change (towards either end of the spectrum) over time. The Laennec staging classification further categorizes cirrhosis into mild, moderate, and severe fibrosis and correlates with clinical outcomes, including decompensation and liver-related death.[10] Since the altered blood flow in cirrhosis is likely due (at least in part) to fibrosis, the degree of fibrosis in cirrhosis may affect the ability of in-transit metastases to access the liver vasculature.[7] Hence, although we do not use Laennec staging in daily diagnostic practice, we employed it for the purposes of this study, hypothesizing that metastases can occur in cirrhotic livers where the fibrous remodeling is not severe. As an extension of this argument, we also hypothesized that abnormal veno-arterial shunts may effectively bypass more severe stages of fibrosis. While there are robust autopsy-based studies examining the prevalence of metastases in cirrhotic livers, studies of metastases in the cirrhotic liver of living patients are essentially limited to colorectal cancer.[6, 11–13] With that in mind, and given that liver masses are a common and critically important specimens in the practice of surgical pathologists, we aim in this study to provide a systematic evaluation of the likelihood that a biopsy or resections of a mass in cirrhotic liver will contain metastatic disease.

## Materials and methods

### Cases and Data Collection

We searched the electronic pathology records at 7 tertiary care institutions for biopsy, excision, or surgical resection specimens of cirrhotic livers with mass lesions. Among these specimens were well-defined examples of metastases to liver. As our control population, we selected cases from surgical pathology archives at these institutions of cirrhotic livers without mass lesions in patients who had histologic confirmation of distant metastases at other sites. Because few such control cases were identified in our surgical pathology archives, autopsy archives were also searched. We also developed separate control populations of patients with primary liver neoplasms and cirrhosis, and patients with masses in non-cirrhotic livers. Patient medical records of cirrhotic metastasis cases were reviewed for clinical history (including possible sequelae of cirrhosis), radiologic data (including echocardiogram and ultrasound Doppler results, which were reviewed for evidence of veno-arterial shunting) and laboratory values. This information was also reviewed in the controls with cirrhosis and metastatic disease that did not involve the liver, and in a subset consisting of 10 % of the controls with primary liver tumors and cirrhosis. The study was approved by the Institutional Review Boards at each of the involved institutions.

### Special Stains and Immunohistochemistry

Routine hematoxylin and eosin and trichrome stains for case and control specimens were reviewed, when available, in all study cases and controls. For those cases where a trichrome stain was not available, new Masson or Gomori trichrome stains were performed according to standard institutional protocols at each study site. Immunohistochemical stains for CD34 were performed on all specimens from cases of metastases in cirrhosis and in the subset of 10 % of primary liver cancers chosen for further clinical review, again using routine protocols at each institution.

### Histologic Review

**Fig. 1:**
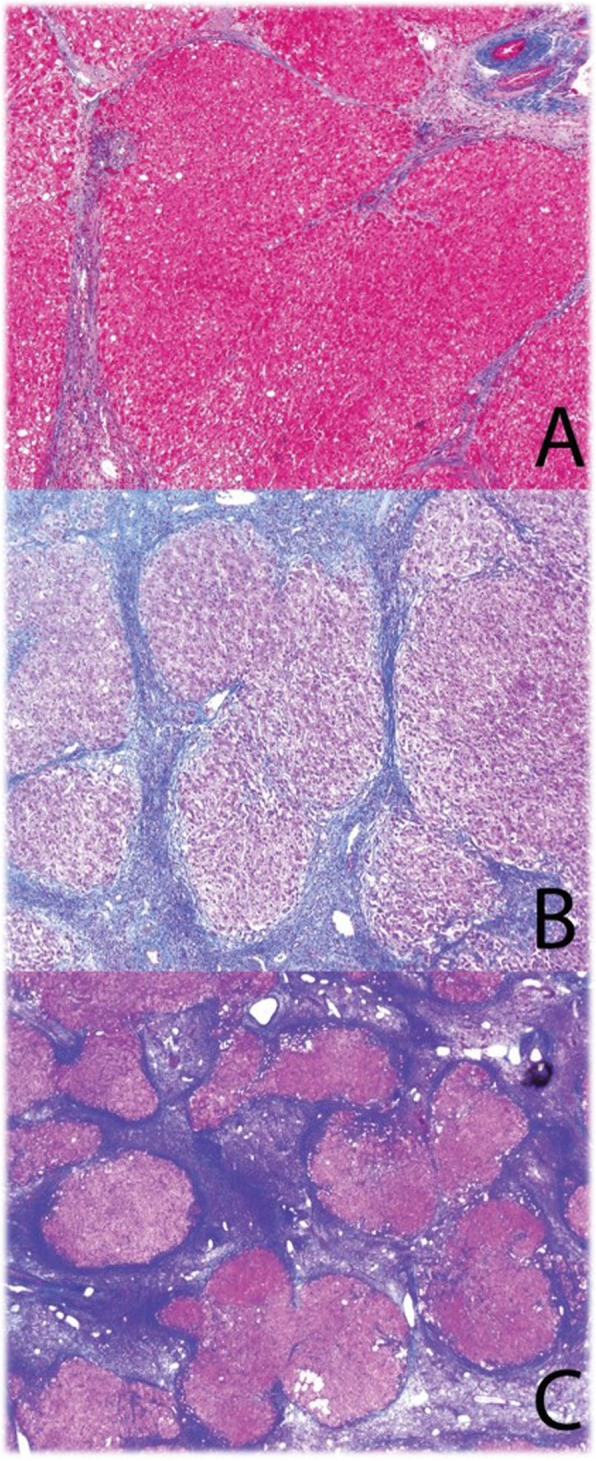
Laennec staging in cirrhosis. **a** Laennec stage 4A is characterized by mild cirrhosis with delicate fibrous septae, trichrome stain. **b** Laennec stage 4B (moderate) cirrhosis with broad fibrous septae, trichrome stain. **c** Laennec 4 C (severe) cirrhosis with very broad septae, trichrome stain.

Cirrhosis was categorized by the Laennec fibrosis staging as described previously: 4A = mild cirrhosis (delicate fibrous septa seen on trichrome stain), 4B = moderate (at least 2 broad septa), 4 C = severe cirrhosis (at least 1 very broad septum or many minute nodules) (Fig. 1).[10] Immunohistochemical staining for aberrant CD34 in the sinusoids in the background cirrhotic liver was considered evidence of sinusoidal capillarization due to severe portal vein obstruction.

## Results

**Fig. 2:**
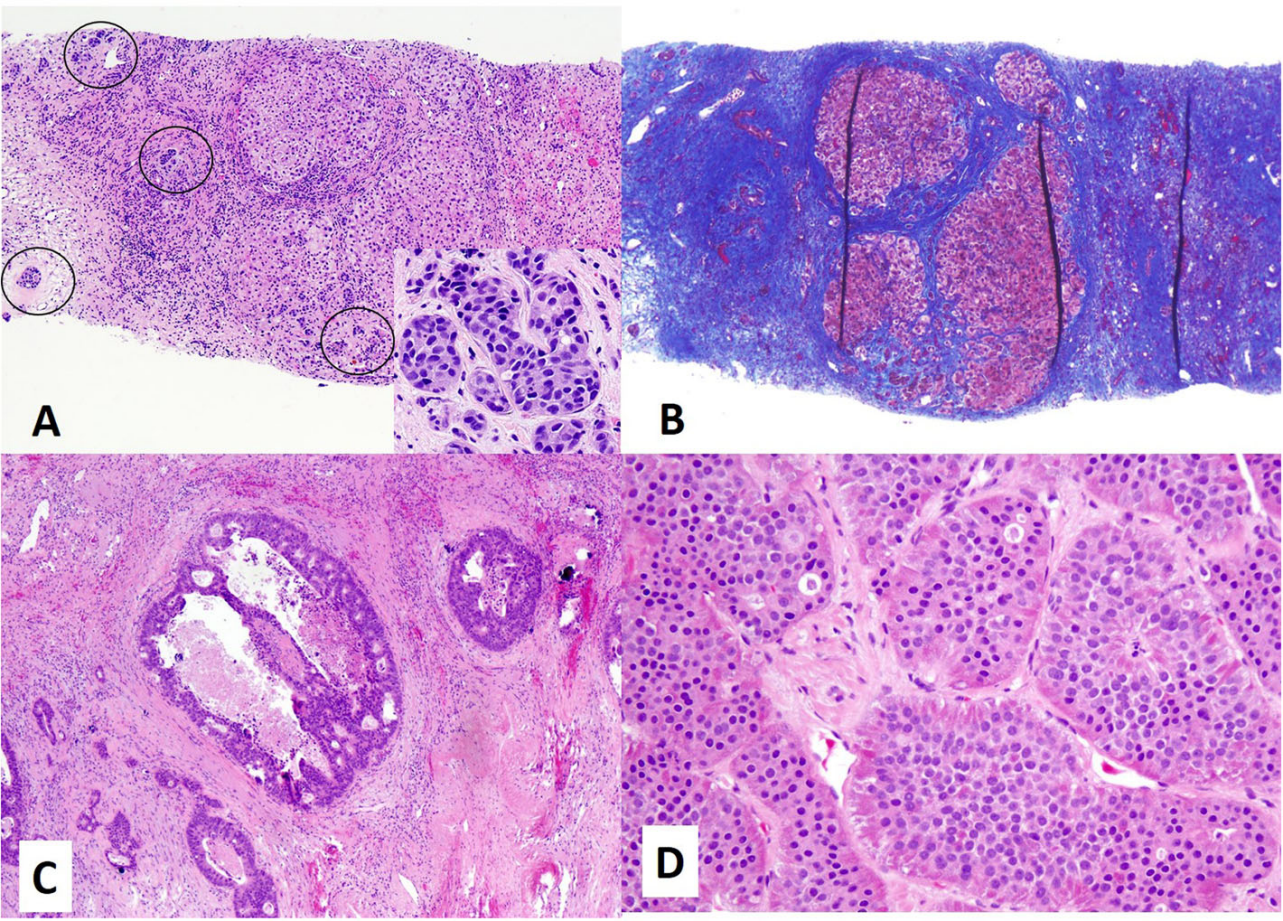
Representative metastases in cirrhotic livers. **a** Metastatic invasive ductal carcinoma of breast, within cirrhotic parenchyma, H&E. The circled areas are the metastatic tumor cells. Inset shows a high power magnification of the carcinoma. **b** Trichrome stain of the case of metastatic invasive ductal carcinoma, demonstrating cirrhosis. **c** Metastatic colorectal adenocarcinoma, H&E. **d** Metastatic well-differentiated neuroendocrine tumor, H&E.

Of the 1453 biopsies, excisions, or resections of masses in cirrhotic livers, 24 (1.7 %) were metastases (Table 1); representative examples of metastases are seen in Fig. 2. The clinicopathologic features of these metastases are summarized in Table 2. The average age of patients with metastases in cirrhotic was 64 years (range 47–78); most (15) patients were women. The most common primary tumor responsible for metastases in cirrhotic livers was colorectal carcinoma (8 cases; 33 %). Other primary tumors included neuroendocrine neoplasms from any site (5; 21 %), as well as carcinomas of pancreas (4; 17 %), upper gastrointestinal tract (2; 8 %), breast (2; 8 %), fallopian tube (1; 4 %) and lung (1; 4 %). A single case (4 %) of carcinoma of unknown primary was included. Neuroendocrine neoplasms were from pancreas (2), small bowel (1), lung (1) or from an unknown site (1).

**Table 1:** Cases of metastases in cirrhotic livers

Case	Age (years)	Sex	Resection or biopsy	Original site of tumor	Cause of cirrhosis	Laennec stage (4A, 4B, 4 C)	AST	ALT	Albumin (g/dL)	Platelets (10^3/µL)	Cirrhosis-related sequalae	Echocardiogram (evidence of shunts or septal defects)	Ultrasound doppler information	CD34 aberrant positivity of background cirrhotic liver
1	68	M	Biopsy	Pancreatic ductal adenocarcinoma	HCV	4B	161	107	3.2	292	None	N/A	Patent main portal vein with no flow reversal	No
2	47	F	Biopsy	Well-differentiated neuroendocrine tumor (small bowel)	EtOH	4A/B	22	25	4.1	195	None	No evidence of shunt	Patent main portal vein with no flow reversal	No
3	69	F	Biopsy	Metastatic undifferentiated carcinoma (history of breast carcinoma)	Steatohepatitis	4 C	721	1166	2.8	230	None	No evidence of shunt	Patent main portal vein with no flow reversal	No
4	65	F	Biopsy	Breast ductal carcinoma	Cryptogenic	4 C	134	83	2.8	220	Ascites; jaundice	N/A	Flow reversal in main portal vein	No
5	66	M	Biopsy	Lung adenocarcinoma (favored)	EtOH	4 C	33	26	3.6	94	Varices	No evidence of shunt	Flow reversal in portal vein and branches	No
6	77	F	Biopsy	Poorly differentiated carcinoma with intestinal differentiation (possibly colonic)	Cryptogenic	4B	92	32	2.5	150	Ascites	No evidence of shunt	N/A	No
7	56	F	Resection	Colorectal adenocarcinoma	HCV	4A/B	60	33	4.4	332	Ascites; collateral vessels	No evidence of shunt	N/A	No
8	67	F	Resection	Colorectal adenocarcinoma	NASH	4A	68	35	2.6	200	None	No evidence of shunt	N/A	No
9	68	F	Biopsy	Small cell carcinoma	History of HCV (status post transplant)	4A	312	155	2.2	86	Ascites; portal hypertension; splenomegaly	No evidence of shunt	N/A	No
10	67	M	Biopsy	Pancreatic ductal adenocarcinoma	HCV	4 C	104	106	4	137	Ascites; varices; portal hypertension	N/A	N/A	N/A
11	59	M	Biopsy	Well-differentiated neuroendocrine tumor (pancreas)	NASH	4A	27	27	4	251	Ascites	No evidence of shunt	N/A	N/A
12	74	F	Biopsy	Colorectal adenocarcinoma	NASH	4 C	14	10	4	74	Ascites; splenomegaly; portal hypertension	No evidence of shunt	Patent main portal vein with no flow reversal	N/A
13	78	M	Biopsy	Colorectal adenocarcinoma	EtOH	4A	721	109	2	82	Ascites; varices; portal hypertension; encephalopathy	N/A	Patent main portal vein with no flow reversal	N/A
14	59	M	Resection	Well-differentiated neuroendocrine tumor (unknown primary)	EtOH	4B	N/A	N/A	3.9	11	Ascites; encephalopathy	N/A	Patent main portal vein with near (but not complete) flow reversal	No
15	48	M	Resection	Colorectal adenocarcinoma	Cryptogenic	4A	47	70	4.6	150	None	N/A	Patent main portal vein with no flow reversal	No
16	51	F	Resection	Pancreatic ductal adenocarcinoma	NASH	4A	88	60	4.3	259	None	N/A	N/A	No
17	50	F	Biopsy	Fallopian tube serous carcinoma	NASH	4A	131	127	3.1	341	None	No evidence of shunt	Patent main portal vein with no flow reversal	N/A
18	65	M	Biopsy	Colorectal adenocarcinoma	HCV	4B	36	44	N/A	77	None	N/A	N/A	N/A
19	60	F	Biopsy	Gastric adenocarcinoma	Steatohepatitis	4 C	81	40	2.3	220	Ascites	No evidence of shunt	Main portal vein with possible nonocclusive thrombus; no flow reversal	N/A
20	73	F	Wedge biopsy	Duodenal adenocarcinoma	HCV	4A	32	22	3.4	119	None	No evidence of shunt	N/A	N/A
21	62	F	Biopsy	Pancreatic ductal adenocarcinoma	Cryptogenic	4A	45	26	2.7	146	Ascities	N/A	N/A	N/A
22	75	F	Biopsy	Metastatic carcinoma of unknown primary	Steatohepatitis	4 C	54	25	2.1	127	Ascites; varices	N/A	N/A	N/A
23	74	M	Biopsy	Colorectal adenocarcinoma	Steatohepatitis	4B	N/A	N/A	N/A	N/A	Unknown	N/A	N/A	No
24	57	F	Biopsy	Pancreatic ductal adenocarcinoma	HCV	4A	34	14	2.4	269	None	No evidence of shunt	N/A	No

**Table 2:** Summary of clinicopathologic features of metastases in cirrhosis

	*n (%)* unless stated otherwise
Sex	
Female	15 (62.5)
Male	9 (37.5)
Age, *mean (range)*	64 (47, 78)
Laennec Stage	
4A	10 (41.7)
4B	7 (29.2)
4 C	7 (29.2)
Primary site	
Colorectal	8 (33)
Neuroendocrine	5 (21)
Pancreas	4 (17)
Upper Gastrointestinal	2 (8)
Breast	2 (8)
Mullerian	1 (4)
Lung	1 (4)
Unknown	1 (4)
AST, *mean ± SD*	137 ± 200
ALT, *mean ± SD*	106 ± 240
Albumin, *mean ± SD*	3.2 ± 0.8
Platelets, *mean ± SD*	177 ± 89
Cirrhosis-related sequelae	13 (54.2)
Echocardiogram with evidence of shunt	0 (0)
Reversal of portal vein flow	2 (18.2)

For comparison, we identified 8 liver samples (including 5 autopsy specimens) in patients who had both cirrhotic livers and malignancies with distant metastases at other sites without involvement of the liver. The average age of patients in this control group was 63 years (range 52–78); 5 of these patients were women. The primary sites in this control population included carcinomas from lung (1), colon (1), fallopian tube (1), breast (1), renal (1), pancreas (1), and thyroid (1), as well as 1 poorly differentiated neoplasm which was thought to be a malignant mesothelioma. We also examined 147 primary liver tumors from cirrhotic livers (138 hepatocellular carcinomas, 9 cholangiocarcinomas). Finally, we identified 29 metastases to non-cirrhotic livers (7 surgical excisions/resections and 22 biopsies). Of these, 5 (3 biopsies, 2 excisions/resections) had fibrosis patterns up to and including bridging fibrosis, insufficient for a diagnosis of cirrhosis. The primary sites of carcinomas in this control group included colon (11), esophagus (3), lung (3), pancreas (2), breast (2), prostate (1), ureter (1), bladder (1), kidney (1), and pituitary (1), Three melanomas were also included.

### Histologic evaluation of metastases in cirrhotic livers and controls

**Fig. 3:**
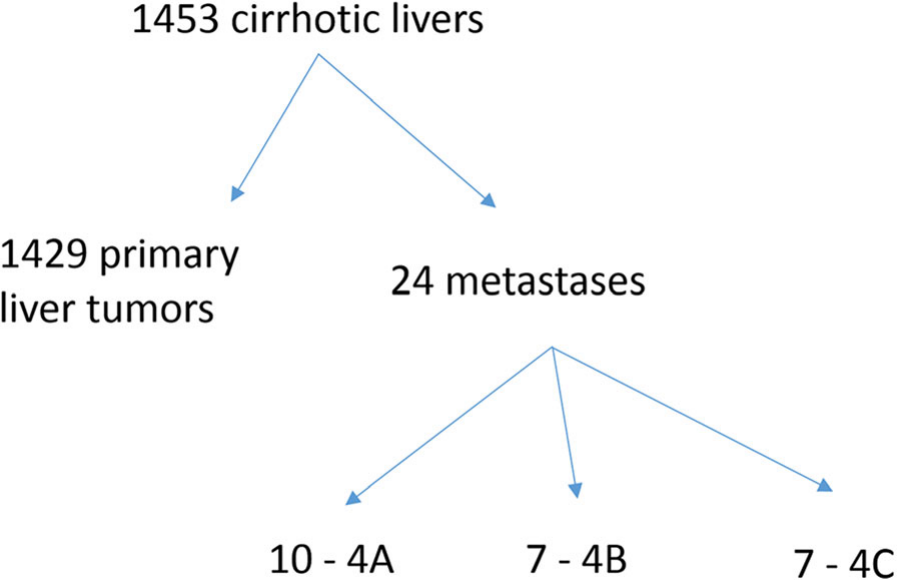
Flowchart of metastases in cirrhotic livers by Laennec stage 4A, 4B and 4 C.

By Laennec staging, most (17 of 24) cirrhotic livers with metastases were 4A (10) or 4B (7), while the remaining 7 livers were Laennec 4 C (Fig. 3). The majority of samples in the control cirrhosis group from patients with malignancies that had metastasized to sites other than liver were also Laennec 4A (3) or 4B (3), while only 2 livers were Laennec 4 C. Of the cirrhotic livers with primary liver tumors, 64 were Laennec 4A and 64 were 4B. The remaining 28 cases were 4 C.

CD34 immunohistochemical tests did not show evidence of generalized sinusoidal capillarization in any cases of metastases in cirrhotic livers. In comparison, 1 of 14 controls with primary liver cancer in cirrhosis had CD34 positivity in the background liver suggestive of generalized sinusoidal capillarization.

### Clinical, radiologic and laboratory data in cirrhotic livers and controls

The causes of cirrhosis in patients with metastases were steatohepatitis (13 total cases of steatohepatitis; 5 of which were non-alcoholic, 4 of which were alcoholic, and 4 of which were due to steatohepatitis not otherwise specified), hepatitis C virus (7 cases), and cryptogenic (4 cases). There was no discernible relation between etiology of cirrhosis and the primary tumor responsible for metastasis; each etiology was seen in association with multiple sites of origin for metastases. Mean albumin values were 3.2 g/dL in patients with metastases and cirrhotic livers, with mildly elevated transaminase levels (mean aspartate aminotransferase (AST) level = 137; mean alanine aminotransferase level (ALT) = 106). Mean platelet count was 177 × 10^3/uL in these patients (Table 1). In the control population of cirrhotic patients with malignancies that metastasized to sites other than the liver, mean albumin values were 2.9 g/dL with mean transaminase values 62 AST and 30 ALT, while average platelet count was 161 × 10^3/uL.

Clinically, 13 of 24 patients with metastases in cirrhotic livers had sequelae of decompensated cirrhosis, including ascites, varices or other evidence of portal hypertension, and jaundice; ascites was the most common sequela and was present in 12 patients. Ultrasound Doppler imaging was available for 11 patients and showed reversal of portal vein flow in 2 of 5 patients with Laennec 4 C cirrhosis, but all 4A and 4B patients had patent portal veins without evidence of reversed flow. In addition, portal vein thrombi were suggested via Doppler or other imaging in 3 additional Laennec 4 C patients; overall only 2 of 7 patients with Laennec 4 C cirrhosis had patent portal veins and normal flow. Echocardiogram results were available for 13 patients, including 4 with Laennec 4 C cirrhosis, and did not show ventricular or atrial septal defects or arteriovenous shunts (Table 1).

In the control population of patients with distant metastases at other sites without involvement of the liver, 5 of 8 patients had sequelae of cirrhosis, including ascites and portal hypertension. Two patients in this group were evaluated by ultrasound Doppler imaging; the portal vein was patent in both and no reverse flow was seen. Echocardiogram results were available for 7 patients, including one patient with Laennec 4 C cirrhosis; one patient had a patent foramen ovale with a low-grade left-right shunt, while the remaining patients had neither ventricular or atrial septal defects nor shunting.

12 of 14 patients with primary liver cancer in cirrhosis had cirrhosis-related sequelae. Of the 10 cases of primary liver cancer in cirrhosis where Doppler data was reviewed, 1 patient had a right to left shunt while the remaining patients had no significant abnormality on imaging.

## Discussion

Our study offers a multifaceted approach to better elucidate the phenomenon of metastasis to the cirrhotic liver. We combined histologic features with vascular dynamics in order to shed light on the physiology of metastasis to the cirrhotic liver and delinieate clinical scenarios in which this uncommon occurrence may be present. We found that even advanced fibrosis is not an absolute barrier to metastatic disease, particularly if portal circulation is intact and if sinusoidal capillarization, as demonstrated by negative CD34 imunostains, is absent. Although normally, pathologists and clinicians may not expect metastases in a cirrhotic liver, we conclude that involvement from extrahepatic primary tumors is still a possibility, particularly in these settings.

Numerous autopsy studies have demonstrated the rarity of metastases in cirrhotic livers, with prevalence ranging 0–2.9 % in these studies, although systematic and complete clinical cohort studies in living patients are largely lacking (Table 3).[1–3, 14–20] Our results confirm that metastases are rare in cirrhotic livers and provide an estimate of the prevalence of this phenomenon from the perspective of the practicing surgical pathologist. Of 1453 biopsies, excisions or resections of cirrhotic livers with mass lesions, only 1.7 % contained metastases from extrahepatic malignancies.

**Table 3:** Previous studies of metastases in patients with cirrhotic livers

Reference	Publication Year	Overall autopsies	Autopsies with cirrhosis	Autopsies with cirrhosis and extrahepatic cancer	Autopsies with metastases in cirrhotic livers
Lisa et al[16]	1942	6,036		22	6
Wallach et al[3]	1953	10,156	441	67	3
Lieber[1]	1957	29,779	1,073	133	6
Gall[14]	1960	1,001	682	108	29
Ruebner et al[19]	1961	23,000	399	54	11
Norkin et al[18]	1962	15,713	1,268	121	37
Goldstein[15]	1969	4,166		31	12
Melato et al[17]	1989	11,752	1,029	160	29
Vanbockrijck et al[20]	1992	2,162	153	30	10
Pereira-Lima et al[2]	2003	7,092	111	6	0
Summary		110,857	5,156	732	143

Interestingly, the distribution of metastases in cirrhosis is similar to that seen in liver metastases as a whole; a nationwide study of all histologically confirmed liver metastases in the Netherlands showed that colorectal carcinoma made up 35 % of overall metastases compared to 33 % in our study.[21] Our study of cirrhotic livers had proportionately more pancreas and upper gastrointestinal primary tumors (38 %) compared to the 18 % of overall metastases in the Netherlands study, although given the relatively small number of metastases in our study it is difficult to say that these results represent a true difference. With our finding that 16 of 24 (67 %) metastases are from primary malignancies other than colorectal carcinoma, we establish that while the colorectum may be the most common single site responsible for metastasis in this setting, most metastases are from primaries outside the colorectum. Thus a wide differential diagnosis is essential in those rare cases where a metastasis is suspected in a cirrhotic liver.

Several rationales have been offered for the rarity of metastases in cirrhotic livers. Since Paget originally offered his seed-and-soil hypothesis, evidence has emerged supporting the metastasis-suppressing role of various factors in the cirrhotic microenvironment. One proposed explanation for the rarity of metastases in cirrhotic livers is that chronic liver disease reduces the function of hepatocyte membrane lectins, which otherwise may promote metastases by recognizing tumor cell surface carbohydrates and adhering to tumor cells.[22–24] Supporting this hypothesis, injection of hepatocyte lectin blocking agents in a mouse model inhibited liver metastases without any effect on number or size of lung metastases.[22] However, blockades of hepatocyte lectins are likely non-specific unless glycosylation patterns of these lectins are unique, and liver sinusoidal endothelial cell lectins have also been show to play a role in tumor cell adherence.[25] Other possible factors in the low prevalence of metastases in patients with cirrhosis include microRNA let-7a cluster involved in regulating the epithelial-mesenchymal transition of colorectal carcinoma, regulation of FasR expression, and matrix metalloproteinase activity.[26] Thus, hepatocytes, sinusoidal cells, and Kupffer cells all may have a role in the “soil” of the cirrhotic microenvironment. As our study shows that metastases still occur in cirrhosis especially in those patients with less severe fibrosis, further research into the effects of cirrhosis on these microenvironmental factors may be worth pursuing.

With respect to Ewing’s hemodynamic hypothesis, the portal venous system is presumed to be the usual route of entry for metastases into the liver. In cirrhosis, architectural distortion and increased vascular resistance in the portal system contribute to portal hypertension. Porto-systemic shunts develop in response to this increase in portal pressure.[7] This set of vascular phenomena is thought to act against a portal route for metastasis of colorectal and upper gastrointestinal carcinomas. Interestingly, most patients in our cohort, including all with Laennec stage 4A and 4B cirrhosis, had patent portal veins, although most patients with Laennec stage 4 C cirrhosis showed portal venous thrombi or reversal of portal flow. Thus, while the severe hemodynamic changes of Laennec 4 C may make metastasis less likely, it is still possible to develop metastasis in the setting of reversed portal blood flow. Given that hepatic artery resistance is decreased and hepatic artery velocity is increased in cirrhosis as a buffer response to increased portal resistance, we suspect that arterial entry may play a role in these cases.[7].

Finally, in addition to hypotheses related to the physiology and microenvironment of the cirrhotic liver, it has been suggested that the rarity of metastases in cirrhosis may ultimately be a consequence of significant comorbidity: patients with both cirrhosis and extrahepatic cancer may simply not live long enough to develop metastases. Co-occurrence of cirrhosis and extrahepatic cancer is rare even without liver metastasis.[1, 3, 16] Furthermore, in one comparison of colorectal carcinoma patients with cirrhosis compared to similar patients without cirrhosis, overall survival was lower in cirrhotic patients despite similar progression-free survival.[27] In our study, both patients with metastases in cirrhosis and the control group of patients with cirrhosis and non-liver metastasis are composed predominantly of patients with Laennec 4A or 4B cirrhosis. While other factors likely contribute to the rarity of metastases in patients with Laennec 4 C cirrhosis, the overall metabolic burden of both severe cirrhosis and metastatic malignancy may also play a role.

One additional possibilility worth considering is the timeline of progression to cirrhosis compared with the rate of progression of metastatic disease. Our data do not indicate any obvious relation between etiology of cirrhosis and the primary site responsible for metastatic disease. However, it is known that some diseases progress to cirrhosis faster than others, while some cancers grow faster or slower than others. Thus, it is possible that some cases of apparent metastases in cirrhotic livers arise when indolent, slow-growing neoplasms metastasize to livers that are non-cirrhotic at the time of metastasis, after which fibrosis progresses to cirrhosis and metastasis is discovered at a later date.

A strength of our study is a new approach to the topic of metastases in cirrhotic livers. It draws on the prevalence and pathologic characteristics of these cases from the perspective of surgical pathologic evaluation rather than autopsies. In addition, because our study design allowed us to both review imaging data and evaluate the CD34 expression patterns in the sinusoidal endothelium of cirrhotic tissue, we were able to use a comprehensive approach to evaluate vascular status of cirrhotic patients with metastases.

Our study does have important limitations. While our analysis provides an overview of cirrhotic metastases in terms of lesions which may be biopsied or resected for pathologic evaluation, our ability to determine overall prevalence and epidemiology is limited by the clinical situations in which samples might be procured. In patients with cirrhosis and multiple metastases in both the liver and other organs, clinicians may opt for a less technically challenging biopsy in another organ, falsely reducing the prevalence of metastases in our study. In addition, the retrospective nature of our analysis and the chart review design limit the availability of certain clinical data, such as imaging, which were not available on all patients. Finally, the evaluation of Laennec stage in some of the core biopsy specimens is limited by the possible confounding effect of the target mass on adjacent liver parenchyma. To help mitigate this influence, cases where mass effect could not be obviously differentiated from cirrhosis were excluded from our study. Notably, in our control group of noncirrhotic patients with metastases, we did not identify any biopsies with sufficient fibrosis to otherwise meet the criteria for cirrhosis, suggesting that ‘mass effect’ likely does not mimic advanced stage liver disease in most cases. We, nonetheless, demonstrate that metastases do occur in the cirrhotic liver, albeit rarely, and display a predilection for the less severe end of the cirrhotic spectrum. Thus, secondary liver involvement by extrahepatic malignancy remains in the differential diagnosis of liver masses from patients with cirrhosis.

## Data Availability

The datasets generated during and/or analysed during the current study are available from the corresponding author on reasonable request.

## Abbreviations

AST: Aspartate aminotransferase
ALT: Alanine aminotransferase

## Code availability

The code used during the current study are available from the corresponding author on reasonable request.
